# Triclustering-based classification of longitudinal data for prognostic prediction: targeting relevant clinical endpoints in amyotrophic lateral sclerosis

**DOI:** 10.1038/s41598-023-33223-x

**Published:** 2023-04-15

**Authors:** Diogo F. Soares, Rui Henriques, Marta Gromicho, Mamede de Carvalho, Sara C. Madeira

**Affiliations:** 1 LASIGE, Faculdade de Ciências, Universidade de Lisboa, Lisbon, Portugal; 2 INESC-ID and Instituto Superior Técnico, Universidade de Lisboa, Lisbon, Portugal; 3 Instituto de Medicina Molecular and Instituto de Fisiologia, Faculdade de Medicina, Universidade de Lisboa, Lisbon, Portugal

**Keywords:** Data mining, Machine learning, Predictive medicine, Software

## Abstract

This work proposes a new class of explainable prognostic models for longitudinal data classification using triclusters. A new temporally constrained triclustering algorithm, termed TCtriCluster, is proposed to comprehensively find informative temporal patterns common to a subset of patients in a subset of features (triclusters), and use them as discriminative features within a state-of-the-art classifier with guarantees of interpretability. The proposed approach further enhances prediction with the potentialities of model explainability by revealing clinically relevant disease progression patterns underlying prognostics, describing features used for classification. The proposed methodology is used in the Amyotrophic Lateral Sclerosis (ALS) Portuguese cohort (N = 1321), providing the first comprehensive assessment of the prognostic limits of five notable clinical endpoints: need for non-invasive ventilation (NIV); need for an auxiliary communication device; need for percutaneous endoscopic gastrostomy (PEG); need for a caregiver; and need for a wheelchair. Triclustering-based predictors outperform state-of-the-art alternatives, being able to predict the need for auxiliary communication device (within 180 days) and the need for PEG (within 90 days) with an AUC above 90%. The approach was validated in clinical practice, supporting healthcare professionals in understanding the link between the highly heterogeneous patterns of ALS disease progression and the prognosis.

## Introduction

Considering longitudinal data, also referred to as multivariate time series data, three-way data, or multivariate trajectory data, triclustering aims to discover patterns that satisfy specific homogeneity and statistical significance criteria. Given the increasing prevalence of three-way data across biomedical and social domains, triclustering—the discovery of patterns (triclusters) within three-way data—is becoming a reference technique to enhance the understanding of complex biological, individual, and societal systems^1^. Clustering is limited to this end since objects (patients) in three-way data domains are typically only meaningfully correlated on subspaces of the overall space (subsets of features), and although biclustering is able to find correlated objects in a subspace of features or temporal patterns for one feature, cannot consider both time and multiple features^2^.

In clinical domains, triclustering has been successfully applied for different ends: health record data analysis, where triclusters can identify groups of patients with correlated clinical features along time; neuroimaging data analysis in which triclusters correspond to enhanced hemodynamic or electrophysiological responses and connectivity patterns between brain regions; multi-omics, where triclusters capture putative regulatory patterns within omic series data; and multivariate physiological signal data analysis, where triclusters capture coherent physiological responses for a group of individuals^1,3,4^. In spite of triclustering relevance for descriptive tasks (knowledge acquisition), its potential in predictive tasks (medical decision) remains considerably untapped^1^.

In this context, grounded on the potentialities of triclustering approaches, we propose a triclustering-based classifier to learn prognostic models from three-way clinical data, which takes advantage of the temporal dependence between the monitored features, and further enhances model explainability by learning an associative model grounded on local temporal patterns (subsets of features with specific values for a subset of patients in a contiguous set of temporal observations during follow-up). To this end, we propose TCtriCluster, a temporally constrained triclustering algorithm able to mine time-contiguous triclusters that extends the state-of-the-art triCluster algorithm^5^, originally proposed by Zhao and Zaki to mine patterns in three-way gene expression data, to cope with three-way heterogeneous clinical data (patient-feature-time data).

As a case study, we target prognostic prediction in Amyotrophic Lateral Sclerosis (ALS) using a large cohort of Portuguese patients, where the triclusters learned from patients’ follow-up data can be interpreted as disease progression patterns. The patterns identifying groups of patients with coherent temporal evolution on a subset of features are then used for prognostic prediction as features in a state-of-the-art classifier. The prognostic models learned using the proposed triclustering-based classifier predict whether a patient will evolve to a target clinical endpoint within a certain time window. We target five clinically relevant endpoints in ALS: (1) need for non-invasive ventilation (NIV), (2) need for an auxiliary communication device, (3) need for percutaneous endoscopic gastrostomy (PEG), (4) need for a caregiver, and (5) need for a wheelchair.

The major contributions of this work are the following:

- A new pattern-centric data transformation from longitudinal data into multivariate temporal features, the triclusters, yielding both descriptive and discriminative qualities for subsequent learning tasks;
- First study in ALS that comprehensively assesses the state-of-the-art predictability limits of different clinical endpoints of interest, using time windows;
- A new triclustering algorithm, termed TCTriCluster, able to find time-contiguous triclusters with constant and additive forms of homogeneity;
- Discriminative patterns of (ALS) disease progression used for prognostic prediction and whose inspection can putatively help to explain prognostics, aiding medical research and practice.

The gathered results are promising, highlighting the potential of the proposed methodology regarding both predictability (outperforming state-of-the-art alternatives) and interpretability. Some limitations should, however, be pinpointed. First, our results primarily focus on the predictive value of follow-up assessments. Nevertheless, the proposed predictors can straightforwardly combine static features with triclustering-based features (as we show at the end). Second, in spite of the large ALS cohort size (N = 1321), collected at the Portuguese ALS center, data from other ALS centers can be used for further validation.

The proposed triclustering-based classifier can be used to learn prognostic models from follow-up data in other diseases, as well as predictive models from three-way data in other domains. The TCtriCluster algorithm can be further used as a standalone tool to mine arbitrarily positioned, overlapping, and temporally constrained triclusters with constant, scaling, and shifting patterns from three-way heterogeneous data.

## Background and related work

ALS is a neurodegenerative disease characterized by weakness and functional disability, with patients presenting with a different phenotype and progression rate. Most of the patients with ALS die from respiratory complications within the first 3–5 years after disease onset. Notwithstanding, some patients are living up to 10 years, while in more severe circumstances, survival can be shortened to 1 year^6^. Recent studies have reported a prevalence of 8-9 cases in 100.000 inhabitants worldwide^7^, in Portugal, the described prevalence is similar^8^.

In the absence of curative treatment, it is essential to promote timely interventions for prolonging survival and improving quality of life. The most important interventions are NIV, with a major positive impact on survival; augmentative communication for preventing social isolation; PEG to keep appropriate nutrition; routine caregiver support for daily life activities and wheelchair regular outings, e.g. for medical appointments^6,9,10^. Clinicians have been using a well-established scale to determine disease progression: the revised ALS Functional Rating Scale (ALSFRS-R)^11^. This scale has specific questions regarding respiratory symptoms, speaking, swallowing, self-care and walking, which are essential to determine the timing of the several interventions. Regarding respiratory function, a number of tests are used to support the decision of NIV initiation.

Due to the high heterogeneity of this disease, the individual prognosis of an ALS patient is challenging. Therefore it is of utmost importance to develop explainable machine learning models, pinpointing the need for approaches to learn explainable disease progression models that clinicians can effectively use for prognostic prediction and timely interventions, with a possible positive impact on survival and quality of life. Recent years have witnessed an increasing awareness of the potentialities of machine learning amongst ALS researchers, leading to several applications to ALS cohort data^12–21^. The great potential of learning stratification models has also shown opportunities for future clinical trials, besides promoting more accurate and trustable predictions by learning group-specific prognostic models^13,22–24^.

In this context, Carreiro et al.^12^ conducted a pioneer study proposing prognostic models to predict the need for NIV in ALS based on clinically defined time windows. More recently, Pires et al.^22^ stratified patients according to their state of disease progression achieving three groups of progressors (slow, neutral and fast), and proposed specialized learning models according to these groups. They further used patient and clinical profiles with promising results^23^. However, none of their studies took into account the temporal progression of the features. Recently, Martins et al. proposed to couple itemset mining with sequential pattern mining to unravel disease presentation and disease progression patterns and used these patterns to predict the need for NIV in ALS patients^25^. Despite their relevant results, they did not consider the contiguity constraint imposed by the temporality of the patient’s follow-up data. Matos et al.^26^ proposed a biclustering-based classifier. Biclustering was used to find groups of patients with coherent values in subsets of clinical features (biclusters), then used as features together with static data. Besides promising, none of this approach also did not take into account the temporal dependence between the features.

In previous work, a preliminary assessment of the role of classic triclustering approaches for predicting ventilation support needs in ALS was undertaken^27^, and, biclusters discovered in the static dimension of data were considered to predict the need for NIV within specific time windows^28^. Differently from these earlier works, our research proposes a novel triclustering approach grounded on temporal contiguity constraints that yield both higher predictability and better explainability.

Complementarily to the above pattern-centric stances, Pancotti et al.^29^ recently applied state-of-the-art deep learning methods to study disease progression in ALS using a publicly available database (PRO-ACT), showing competitive performance.

Despite the extent of research on ALS prognostic ends, most of the existing works focus on survival prediction, NIV needs, or general changes to the ALS functional rating scale (ALSFRS-R), generally neglecting specific clinical endpoints of interest. Specific clinical endpoints, such as the need for a wheelchair or percutaneous endoscopic gastrostomy, have been primarily studied under descriptive stances, including the analysis of cumulative time-dependent risks^30^. To our knowledge, their predictability under the machine learning stance using time windows and explainable progression patterns remains unassessed.

## Methods

**Figure 1:**
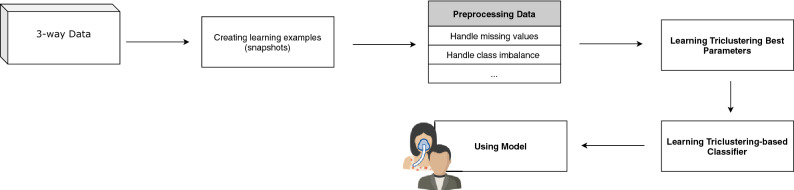
Proposed Workflow to Learn a Triclustering-based Classifier.

This section describes the proposed methodology to learn a triclustering-based classifier from three-way data, from preprocessing (including creating learning examples) to classifier performance evaluation. It further describes TCtriCluster, the proposed triclustering algorithm to mine temporally constrained triclusters. Figure 1 depicts the overall workflow.

In what follows, consider that a three-way dataset, *D*, is defined by *n* objects $X = \{x_1,\ldots ,x_n\}$, *m* features $Y = \{y_1,\ldots ,y_m\}$, and *p* contexts $Z = \{z_1,\ldots ,z_p\}$, where the elements $d_{ijk}$ relate object $x_i$, feature $y_j$, and context $z_k$. Consider also that, a bicluster $B = (I, J)$ is a subspace given by a subset of objects, $I \subseteq X$, and a subset of features, $J \subseteq Y$^2^. Similarly, a tricluster ${\mathscr {T}} = (I, J, K)$, contains $I \subseteq X$ objects, $J \subseteq Y$ features and $K \subseteq Z$ contexts, and $t_{ijk}$ denote the elements of ${\mathscr {T}}$, where $1 \le i \le |I|$, $1 \le j \le |J|$ and $1 \le k \le |K|$^1^. In this context, each tricluster ${\mathscr {T}}$ can be represented as a set of biclusters ${\mathscr {T}} = \{{\mathscr {B}}_1, {\mathscr {B}}_2, \ldots , {\mathscr {B}}_s\}$:

$$\begin{aligned} {\mathscr {B}}_1= & {} \begin{bmatrix} t_{111} &{} t_{121} &{} \cdots &{} t_{1|J|1} \\ t_{211} &{} t_{221} &{} \cdots &{} t_{2|J|1} \\ \vdots &{} \vdots &{} \ddots &{} \vdots \\ t_{|I|11} &{} t_{|I|21} &{} \cdots &{} t_{|I||J|1} \\ \end{bmatrix}, {\mathscr {B}}_2 = \begin{bmatrix} t_{112} &{} t_{122} &{} \cdots &{} t_{1|J|2} \\ t_{212} &{} t_{222} &{} \cdots &{} t_{2|J|2} \\ \vdots &{} \vdots &{} \ddots &{} \vdots \\ t_{|I|12} &{} t_{|I|22} &{} \cdots &{} t_{|I||J|2} \\ \end{bmatrix}, \ldots , {\mathscr {B}}_{|K|} = \begin{bmatrix} t_{11|K|} &{} t_{12|K|} &{} \cdots &{} t_{1|J||K|} \\ t_{21|K|} &{} t_{12|K|} &{} \cdots &{} t_{1|J||K|} \\ \vdots &{} \vdots &{} \ddots &{} \vdots \\ t_{|I|1|K|} &{} t_{|I|2|K|} &{} \cdots &{} t_{|I||J||K|} \\ \end{bmatrix} \end{aligned}$$

### Preprocessing data

The three-way dataset, composed of several heterogeneous features measured over a number of time points, is first preprocessed to obtain learning examples. Depending on the dataset, dealing with missing values and class imbalance might also be needed. Some triclustering searches, such as the one proposed in this work, can ignore missing values, tackling imputation needs.

### TCtriCluster: a new temporal triclustering algorithm

triCluster^5^, a pioneer and highly cited triclustering approach proposed and implemented by Zhao and Zaki is selected. It is a quasi-exhaustive approach, able to mine arbitrarily positioned and overlapping triclusters with constant, scaling, and shifting patterns from three-way data. Given that triCluster was proposed to mine coherent triclusters in three-way gene expression data (gene-sample-time), at this point, it is important to understand that clinical data can be preprocessed in order to have a similar structure, in which patient-feature-time data resembles the gene-sample-time data considered in earlier works. triCluster is composed of 3 main steps: (1) constructs a multigraph with similar value ranges between all pairs of samples; (2) mines maximal biclusters from the multigraph formed for each time point (slices of the 3D dataset); and (3) extracts triclusters by merging similar biclusters from different time points. Optionally, it can delete or merge triclusters according to the placed overlapping criteria.

As our goal is to mine temporal three-way data, meaning the *Z* context dimension corresponds to time, we borrow a pivotal idea behind CCC-Biclustering^31^, a state-of-the-art and highly efficient temporal biclustering algorithm, and introduce a temporal constraint in triclustering to promote interpretability, predictive accuracy, and efficiency. The goal thus becomes to mine Time-Contiguous Triclusters (TCTriclusters), triclusters with consecutive time points. In this context, we re-implemented triCluster in Python and extended it to cope with a time constraint. The new TCtriCluster algorithm implements this time constraint on its 3rd phase, as shown in Algorithm 1 (line 9).

TCtriCluster allows different combinations of input parameters (from the input parameters of triCluster^5^ that should be explored in order to discover the best parameters with which the final classifier should be learned. The input parameters are: $\varepsilon , mx, my, mz, \delta ^x, \delta ^y, \delta ^z, \eta$ and $\gamma$, corresponding to maximum ratio value, the minimum size of tricluster dimensions *x*, *y* and *z*, maximum range threshold along dimensions *x*, *y* and *z*, overlapping and merging threshold, respectively. More details about the input parameters are referred to^5^.
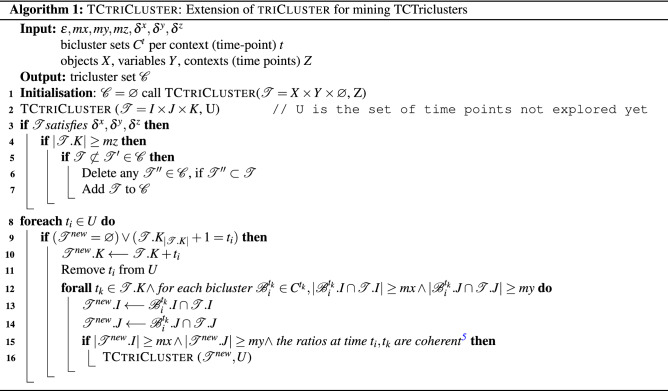


### Hyperparameterizing the triclustering search

**Figure 2:**
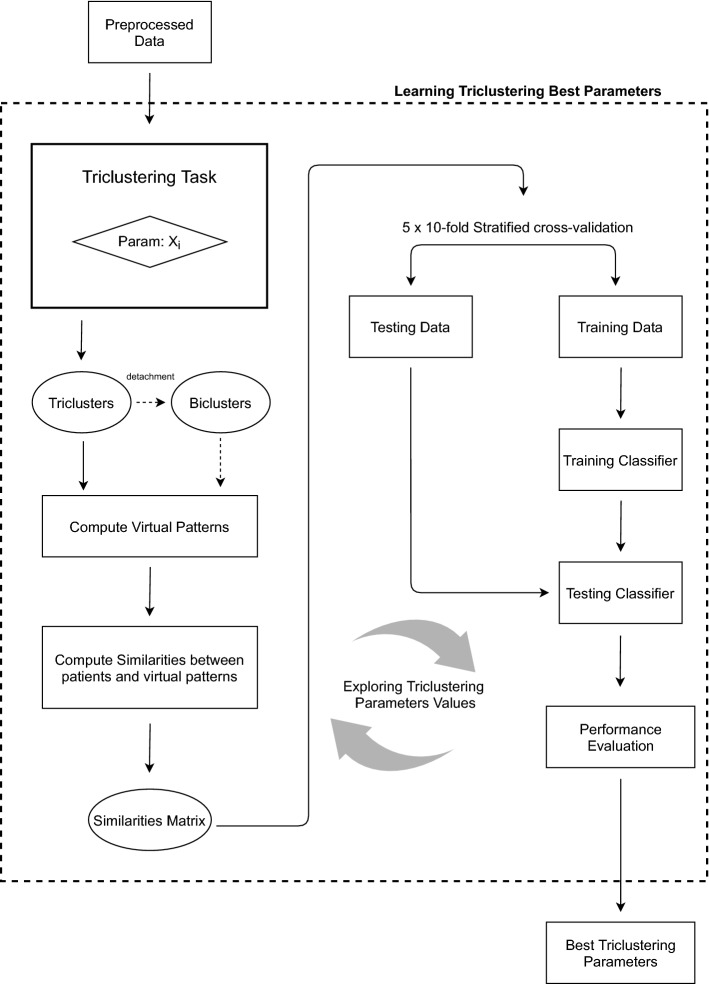
Learning triclustering best parameters: workflow.

In this step, we find the best hyperparameters used as input by the triclustering algorithm (described above) in order to optimize predictive performance. The workflow, depicted in Fig. 2, starts by performing triclustering on the preprocessed data to obtain triclusters. Next, and since our triclustering-based classifier uses the triclusters as features, we compute a 3D virtual pattern for each tricluster.

The proposed 3D virtual pattern corresponds to the tricluster most representative pattern, an extension of the 2D version defined in^32^, and is computed as follows.

#### Definition 1

(*3D virtual pattern*). Given a tricluster ${\mathscr {T}}$, its virtual pattern ${\mathscr {P}}$ is defined as a set of elements ${\mathscr {P}} = \{ \rho _1, \rho _2,\ldots , \rho _{|I|}\}$, where $\rho _i, 1 \le i \le |I|$ is defined as the mean (or the mode, in case of categorical features) of values in the $i^{th}$ row for each context:

1 $$\begin{aligned} \rho _i = \frac{1}{|J|\times |K|} \sum _{z_k\in K} \sum _{y_j\in J} b_{ijk}. \end{aligned}$$

Considering as example a tricluster ${\mathscr {T}}$=(*I*, *J*, *K*), mined from three-way data, (*X*, *Y*, *Z*), composed by 3 objects, 3 features ($y_1$ and $y_7$ are categorical features) and 3 contexts, such that $I = \{ x_1, x_3, x_7 \},\; J = \{ y_1, y_3, y_7 \},\; K=\{ z_2, z_3, z_4 \}$. For simplicity, consider ${\mathscr {T}} = \{ B_2, B_3, B_4 \}$:

$$\begin{aligned}B_2 = \begin{bmatrix} 1 &{} 3.1 &{} 5 \\ 1 &{} 2.8 &{} 3 \\ 3 &{} 2.1 &{} 10 \end{bmatrix}, B_3 = \begin{bmatrix} 2 &{} 3.0 &{} 3 \\ 3 &{} 2.8 &{} 3 \\ 3 &{} 2.9 &{} 9 \end{bmatrix}, B_4 = \begin{bmatrix} 3 &{} 2.9 &{} 3 \\ 2 &{} 2.9 &{} 3 \\ 3 &{} 2.4 &{} 8 \end{bmatrix} \end{aligned}$$

and an object (patient) $P(X_p, I, K)$ defined as $$P = \{ C_2, C_3, C_4 \}: C_2 = \begin{bmatrix} 1&2.22&5 \end{bmatrix}; \; C_3 = \begin{bmatrix} 1&2.26&7 \end{bmatrix}; \; C_4 = \begin{bmatrix} 2&2.35&8 \end{bmatrix}$$. In this settings, the Virtual Patterns are: $$\rho (B_2) = \begin{bmatrix} 1&2.6667&5 \end{bmatrix}$$; $$\rho (B_3) = \begin{bmatrix} 3&2.9&3 \end{bmatrix}$$; $$\rho (B_4) = \begin{bmatrix} 3&2.7333&3 \end{bmatrix}$$; and $$\rho ({\mathscr {T}}) = \begin{bmatrix} 3&2.7667&3 \end{bmatrix}$$.

Note that, optionally, in cases where triclustering could capture heterogeneous triclusters, we can detach the biclusters which compose the tricluster and use those biclusters as features (computing virtual pattern 2D) instead of the pattern that describe the whole tricluster. Notice that in this previous example, if we detached the tricluster, we will use three patterns—$\rho (B_2)$, $\rho (B_3)$ and $\rho (B_4)$—in which the first one is far different from the two others. This optional step gives more information to the classifier, promoting its predictive performance.

With the virtual patterns computed, to assess how well a specific object (patient), $p_i$, follows the general tendency of a given tricluster ${\mathscr {T}}$ we have to compare $p_i$ with the 3D virtual pattern, ${\mathscr {P}}$, which is the most representative pattern of the tricluster ${\mathscr {T}}$. To do this, we propose two approaches: (1) compute the Euclidean distance; or (2) compute Pearson correlation between the 3D virtual pattern ${\mathscr {P}}$ and the equivalent pattern (same features and contexts) of $p_i$.

We denote these assessments as Virtual Distance 3D and Virtual Correlation 3D, and define them as follows:

#### Definition 2

(*Virtual distance 3D*). The virtual Euclidean distance between an observation $p_i$ and a tricluster ${\mathscr {T}}$ is defined as

2 $$\begin{aligned} \texttt {VD}_{\text {3D}}(p_i, {\mathscr {T}}) = E (p_i, \rho ) = \sqrt{\sum ^I_{e=1} (p_{i_e} - \rho _e)^2 }. \end{aligned}$$

#### Definition 3

(*Virtual correlation 3D*). The virtual linear correlation between an object $p_i$ and a tricluster ${\mathscr {T}}$ is defined as

3 $$\begin{aligned} \texttt {VC}_{\text {3D}}(p_i, {\mathscr {T}}) = r (p_i, \rho ) = \dfrac{\displaystyle \sum ^{I}_{e=1} (p_{i_e} - \bar{p_i}) (\rho _e - {\bar{\rho }}) }{\sqrt{\displaystyle \sum ^{I}_{e=1} (p_{i_e} - \bar{p_i})^2 \sum ^{I}_{e=1} (\rho _e - {\bar{\rho }})^2}}. \end{aligned}$$

After computing similarities matrices based on the virtual patterns (using distances or correlations), these matrices are used as learning examples by the classifier (having the triclusters as features) and evaluated with a 5$\times$10-fold Stratified Cross-Validation in order to find the best triclustering parameters, using classification performance as metric. The best parameters are then fed to the next step.

**Figure 3:**
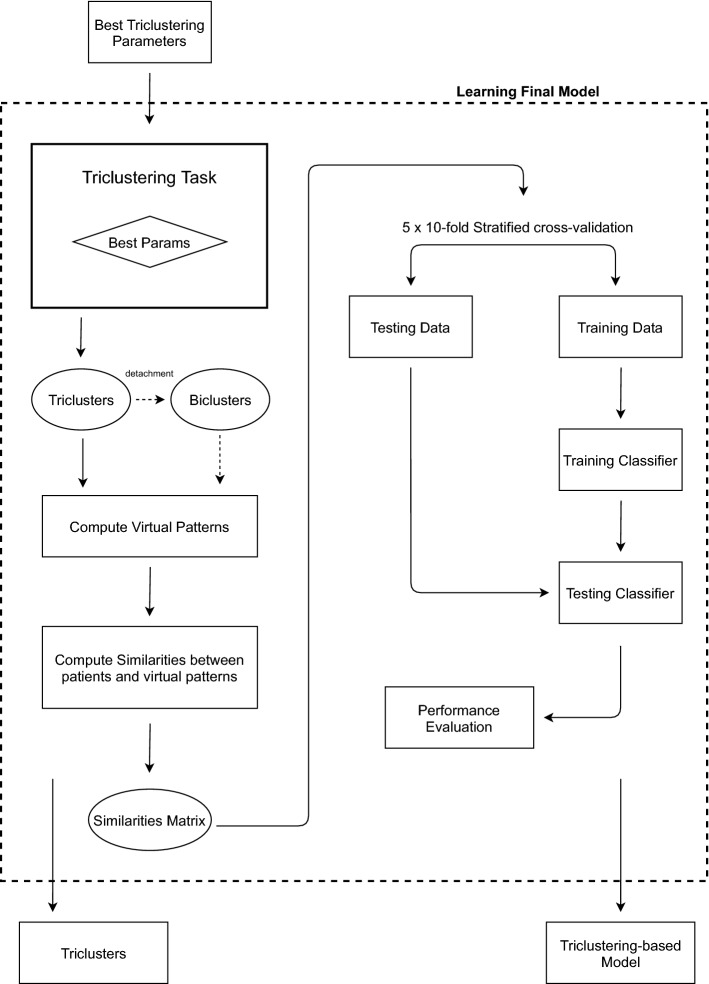
Learning final triclustering-based model: workflow.

### Learning the final classifier

Figure 3 depicts the steps involved in learning the final model. With the best parameters found in the previous step, an additional iteration is performed in order to obtain the final triclusters. The final triclusters are then used to create a classic multivariate data space by creating one variable per tricluster and computing the virtual distance/correlation between each training object and the given tricluster to produce the transformed data. Using this multivariate data space, a traditional classifier can be learned and used to make predictions in the next step.

### Testing stage

**Figure 4:**
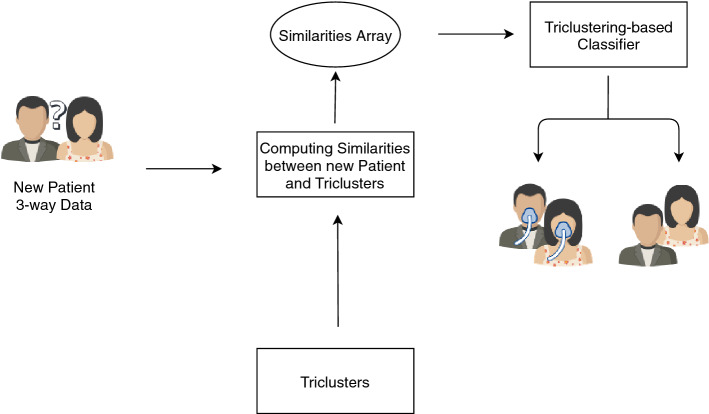
Example of using the triclustering-based classifier to classify new 3-way example from patient follow-up.

After learning the target triclustering-based predictive model, new three-way objects can be classified. To do this, it is necessary to first calculate the array of similarities between the new object and the triclusters (virtual patterns) obtained in the previous steps. This array will be fed to the classifier that will, in turn return the classification for the new object with a percentage of accuracy. Figure 4 depicts an example using clinical three-way data (case study described in the next section).

### Ethics approval and consent to participate

The study was conducted in accordance with the Declaration of Helsinki and was approved by the local (Faculty of Medicine, University of Lisbon) ethics committee. Informed consent to participate in the study was obtained from all participants. Data access was granted in the context of project AIpALS (PTDC/CCI-CIF/4613/2020), where the authors’ institutions participate.

## Case study: prognostic prediction in ALS

In this study, we want to predict whether a given patient will evolve to a critical endpoint within *k* days (time window) since the last clinical appointment using data from the patients’ follow-up. The target endpoints considered and validated by the clinicians are the following:

- C1—need for non-invasive ventilation (NIV), as decided by the international guidelines^11^
- C2—need for an auxiliary communication device (question 1 of the ALSFRS-R with a score of 1 or lower)
- C3—need for percutaneous endoscopic gastrostomy (PEG) (question 3 of the ALSFRS-R with a score of 2 or lower)
- C4—need for a caregiver (question 5 or 6 of the ALSFRS-R with a score of 1 or lower)
- C5—need for a wheelchair (question 8 of the ALSFRS-R with a score of 1 or lower)

**Figure 5:**
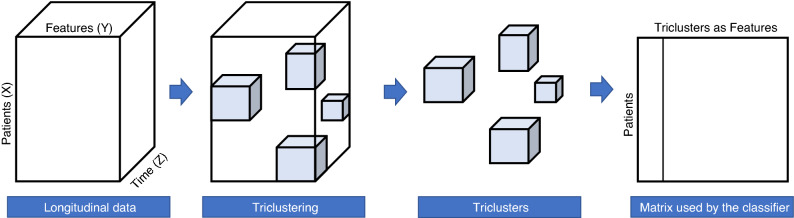
Overview of triclustering-based classifier applied to ALS case study. Three-way data corresponds to longitudinal data collected at patients’ follow-up, and in particular, the dimensions X, Y, and Z correspond to patients, features and time.

In order to apply the triclustering-based classification approach, the three-way data corresponds to longitudinal data collected at the patient’s follow-up, and in particular, the dimensions X, Y, and Z correspond to patients, features, and time, as shown in Fig. 5.

### Cohort data

Our study is conducted using the Lisbon ALS clinic dataset containing Electronic Health Records from ALS Patients regularly followed at the local ALS clinic since 1995 and last updated in October 2021. Its current version contains 1321 patients (740 males and 581 females) with age at onset average $63 \pm 13$ years. Each patient incorporates a set of static features (demographics, disease severity, co-morbidities, medication, genetic information, exercise, and smoking habits, past trauma/surgery, and occupations) along with temporal features (collected repeatedly at follow-up), like disease progression tests (ALSFRS-R scale, respiratory tests, etc.). Table 1 shows the patient cohort characterization.

As the proposed methodology is focused on three-way clinical data analysis and in order to test its potential, we first restrict our data to temporal data only, discarding static data (described in Table 1). We considered 7 features per time point, the Functional Scores (ALSFRS-R), briefly described next, and a respiratory test: Forced Vital Capacity (FVC). Following recent studies^33,34^, we computed an extra temporal feature based on ALSFRS-R scale: MITOS stage^33^. The values for this feature range between 0-5 and provides information about the patient’s disease stage at the moment of the assessment. Concretely, the value represents the number of compromised ALSFRS-R domains^33–35^. The value 5 represents death.

**Table 1:** Characterization of the population used in the case study.

	N = 1321	
Gender		
Male	740	56.0%
Female	581	44.0%
Onset		
Spinal	856	64.8%
Bulbar	348	26.3%
Respiratory	40	3.0%
Axial	28	2.1%
Generalized	39	3.0%
FTD	10	0.8%
Revised El Escorial		
Definitive	231	17.5%
Probable	680	51.5%
Possible	86	6.5%
PMA	190	14.4%
PLS	4	0.3%
NA	130	9.8%
Family history		
Yes	95	7.2%
No	1143	86.5%
NA	83	6.3%
C9orf72 HRE		
Yes	40	3.0%
No	461	34.9%
Unknown	820	62.1%
Age at onset (years)		
Median, IQR	64	55–72
Average, Std	62.6	12.5
Diagnostic delay (months)		
Median, IQR	12	7.5−20
Average, Std	18.1	21.5
BMI at diagnosis (kg/m^2^)		
Median, IQR	24.5	22.4–27.1
Average, Std	24.85	3.8

ALSFRS-R scores for disease progression rating are an aggregation of integers on a scale of 0 to 4 (where 0 is the worst and 4 is the best), providing different evaluations of the patient functional abilities at a given time point^35^. This functional evaluation is based on 12 questions, explained in Table   2. Different functional scores are then computed using subsets of scores, as shown in Table 3.

**Table 2:** ALSFRS-R questions.

Q1—Speech	
Q2—Salivation	
Q3—Swallowing	
Q4—Handwriting	
Q5—Cutting food and Handling Utensils	
Q6—Dressing and Hygiene	
Q7—Turning bed and adjusting bed clothes	
Q8—Walking	
Q9—Climbing Stairs	
Q10—Dyspnea	
Q11—Orthopnea	
Q12—Respiratory Insufficiency

**Table 3:** Functional scores and sub-scores according to ALSFRS-R.

Functional score	Description
ALSFRS-R (total score)	Sum of Q1–Q12
ALSFRSb	Q1 + Q2 + Q3
ALSFRSsUL	Q4 + Q5 + Q6
ALSFRSsLL	Q7 + Q8 + Q9
R	Q10 + Q11 + Q12

### Preprocessing

Data were preprocessed in accordance with the approach proposed by Carreiro et al.^12^, which assumes the patients are followed up regularly and perform a normative set of tests after each appointment. As patients may not be able to perform all tests in a single day, the method takes their temporal distribution into account when learning from the available clinical records by computing snapshots of the patient’s condition by grouping tests performed within a clinically accepted time window.

Following these assumptions, we performed a hierarchical (agglomerative) clustering with constraints to compute the patient’s snapshots, a state-of-the-art procedure to perform alignments along a follow-up^12^. The constraints applied when grouping the sets of evaluations followed well-established principles as in^12^: (1) the evaluations that compose a snapshot cannot belong to the same test as clinicians do not prescribe the same test twice; and (2) all the evaluations considered in the same snapshot should be consistent regarding the critical features of interest (i.e., the patient should be either in the critical endpoint or not in all the records composing the snapshot). For this study, the cutting point for creating the snapshots was defined as 100 days and goes in line with Carreiro et al.^12^.

**Figure 6:**
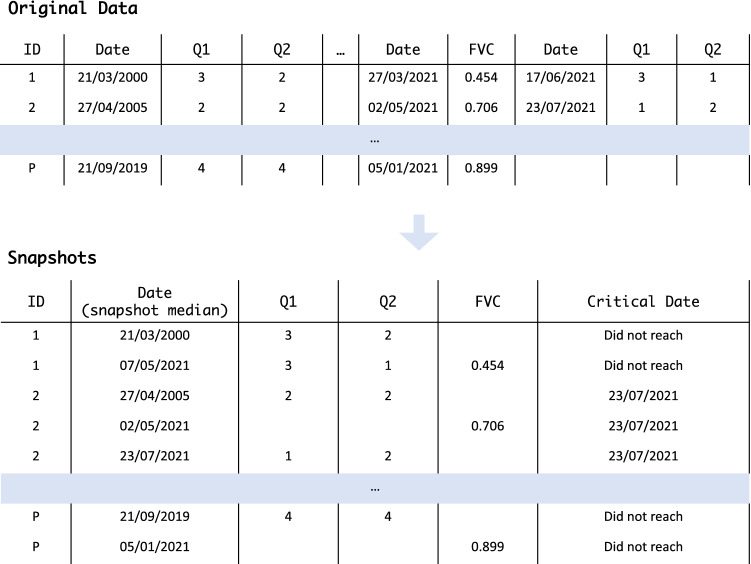
An example of the transformation of the original data into patient snapshots following Carreiro et al. approach^12^. Patient 2 is the only individual who reached a C2 critical status (Q1 $\le$ 1), with the corresponding date being identified in its snapshots. Other critical dates based on tests are further computed based on well-established clinical criteria.

At this stage, we compute five datasets (one for each of the critical endpoints) with the patient’s snapshots which have a critical feature, establishing, for each snapshot, if the patient is or is not in a critical endpoint (binary feature). The critical feature value (target to be learned by the classifier) was computed for each critical endpoint based on the date on which a patient’s critical status was detected. For each one, the critical date considered and validated by the clinicians was the date of the first evaluation with the following ALSFRS-R conditions (see Table 2):

- C1: critical when Q12 $$\le$$ 3
- C2: critical when Q1 $$\le$$ 1
- C3: critical when Q3 $$\le$$ 2
- C4: critical when Q5 $$\le$$ 1 $$\vee$$ Q6 $$\le$$ 1
- C5: critical when Q8 $$\le$$ 1

As an example, for the target endpoint C1 (need for NIV), the critical feature identifies whether a patient will evolve to a critical status (need for NIV), occurring when the patient has a date within the defined interval where the Q12 score is lower than 3. Figure 6 depicts an example of the computation of patient snapshots.

After creating the patients’ snapshots, we have to compute the learning examples used by the predictive models. According to its critical point of interest, each dataset needs to have the patient’s evolution for a critical state, depending on the observed changes *k* days from the snapshot. We create the binary target class *Evolution (E)*, where 1 represents an evolution for a critical status within *k* days from the snapshot, and 0 represents an unchanged critical status within the same time window.

**Figure 7:**
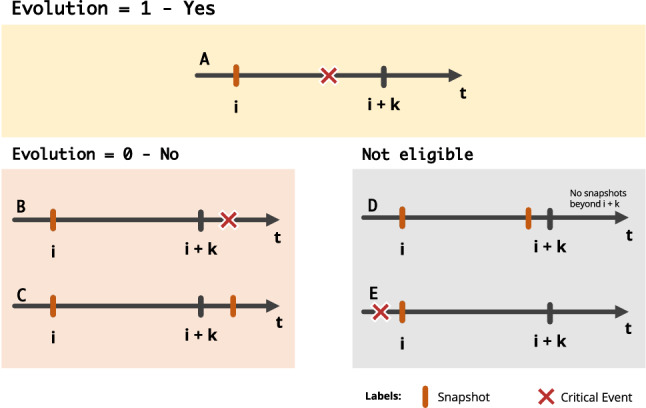
Definition of class Evolution (E) according to the patient’s evolution to a critical status in the interval of *k* days where *i* is the median date of the snapshot.

The process of labelling the snapshots is performed based on the date on which a critical status was detected^12^. A patient’s snapshot (with date *i*) in which he/she was in a critical state between *i* and $i + k$ is labelled as *E=1* (situation A). The snapshots having a date more than *k* days before the critical status date (outside the time window) are labelled as *E=0* (situation B). In the case of patients for who a crtical status has never been detected, their snapshots are labelled as *E=0*, existing at least one snapshot after $i + k$ days (situation C). The snapshots with no critical status information after $i + k$ days are considered not eligible for the analysis since it is impossible to ensure an evolution or not to a critical status in the considered time window (situation D). The snapshots in which the patient is in a critical status are also not eligible for the analysis since we aim to predict the evolution from a non-critical state to a possible critical one (situation E). Figure 7 shows examples of the Evolution computing process.

We chose 3 clinically relevant time windows for this study: 90, 180 and 365 days (3, 6 and 12 months). Therefore, the process resulted in 3 datasets for each target endpoint and time window (15 in total). The number of snapshots in each dataset (discriminated by the classes) is documented in Table 4.

**Table 4:** Initial class distribution concerning each critical endpoint of interest and time windows (after snapshots creation)—N is the number of snapshots in which the patient will not evolve within the considered time window since the date of the snapshot, and Y is the number of snapshots in which the patient will evolve.

	90 days		180 days		365 days	
	N	Y	N	Y	N	Y
**C1**	3803 (96%)	176 (4%)	3315 (83%)	664 (17%)	2693 (68%)	1286 (32%)
**C2**	4845 (98%)	117 (2%)	4574 (92%)	388 (8%)	4193 (85%)	769 (15%)
**C3**	5548 (99%)	60 (1%)	5358 (96%)	250 (4%)	5031 (90%)	577 (10%)
**C4**	2519 (93%)	190 (7%)	2072 (76%)	637 (24%)	1513 (56%)	1196 (44%)
**C5**	4593 (97%)	125 (3%)	4208 (89%)	510 (11%)	3583 (76%)	1135 (24%)

C1, need for NIV; C2, need for an auxiliary communication device; C3, need for PEG; C4, need for a caregiver and C5, need for a wheelchair.

**Figure 8:**
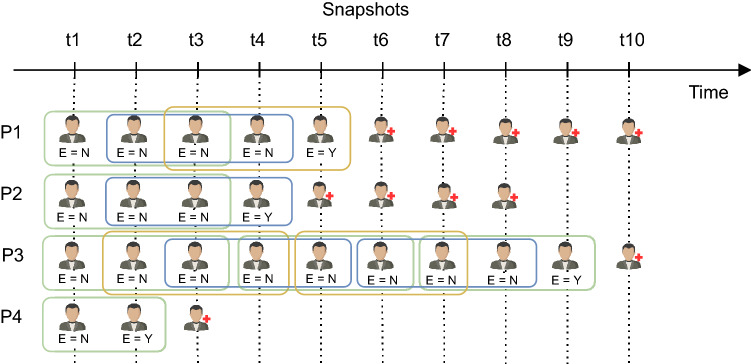
Example on the computation of snapshots with maximum length $\min (L, nP)$, in this case, L = 3 and *nP* is represented by the number of snapshots (where the patient was not in a critical state) availabe for each patient. P4 has only 2 ($nP=2$) snapshots before the critical state, and only one set with these 2 snapshots was considered.

Finally, since the underlying triclustering algorithm is a quasi-exhaustive algorithm^1^ and we want to make the predictions based on current and recent clinical evaluations, we defined a maximum length on historical data to assist the prognostic tasks. With this assumption, we need to transform our datasets coupling snapshots to create the final learning instances which will feed to the model. The process of grouping snapshots is depicted in Fig. 8 and consists in defining a maximum size *L* and grouping consecutive snapshots for each patient. The size of sets (number of snapshots) will be defined by $\min (L, nP)$ where *nP* is the number of available snapshots for a given patient.

The final learning examples, used in the experiments, considered 3, 4, and 5 consecutive snapshots (CS) per patient, corresponding to clinical evaluations at 3, 4, and 5 consecutive appointments, respectively. The Evolution (Y or N) label of the last snapshot is considered as the target class. The new class distributions and the coupled snapshots are depicted in Table 5.

**Table 5:** Initial class distribution concerning each target endpoint and time window, after creating the learning examples considering 3, 4, and 5 consecutive snapshots of patient historical assessments.

		90 days		180 days		365 days	
		N	Y	N	Y	N	Y
**C1**	3 CS	2640 (94%)	176 (6%)	2228 (83%)	445 (17%)	1868 (85%)	331 (15%)
	4 CS	2229 (93%)	176 (7%)	1839 (81%)	423 (19%)	1571 (85%)	282 (15%)
	5 CS	1912 (92%)	175 (8%)	1537 (79%)	408 (21%)	1338 (84%)	255 (16%)
**C2**	3 CS	3533 (97%)	128 (3%)	3304 (92%)	285 (8%)	3093 (94%)	199 (6%)
	4 CS	3045 (96%)	127 (4%)	2822 (91%)	279 (9%)	2668 (94%)	176 (6%)
	5 CS	2647 (95%)	127 (5%)	2434 (90%)	269 (10%)	2317 (94%)	159 (6%)
**C3**	3 CS	4058 (98%)	62 (2%)	3888 (96%)	183 (4%)	3669 (97%)	126 (3%)
	4 CS	3474 (98%)	62 (2%)	3308 (95%)	179 (5%)	3144 (97%)	97 (3%)
	5 CS	3001 (98%)	62 (2%)	2846 (94%)	168 (6%)	2721 (97%)	75 (3%)
**C4**	3 CS	1692 (87%)	263 (13%)	1298 (71%)	523 (29%)	1005 (72%)	388 (28%)
	4 CS	1415 (84%)	263 (16%)	1041 (67%)	504 (33%)	833 (70%)	356 (30%)
	5 CS	1226 (82%)	263 (18%)	866 (64%)	490 (36%)	714 (68%)	329 (32%)
**C5**	3 CS	3246 (96%)	146 (4%)	2907 (88%)	398 (12%)	2479 (89%)	309 (11%)
	4 CS	2730 (95%)	146 (5%)	2402 (86%)	387 (14%)	2066 (89%)	266 (11%)
	5 CS	2328 (94%)	146 (6%)	2009 (84%)	378 (16%)	1750 (89%)	223 (11%)

C1, need for NIV; C2, need for an auxiliary communication device; C3, need for PEG; C4, need for a caregiver, and C5, need for a wheelchair.

Table 5 shows we face considerable class imbalance. In some time windows considered in this case study, the expression of non-evolution patients (class N) is far superior to that of evolution patients (class Y). To tackle this evident imbalance and prevent its drawbacks in the classification process, when the number of examples belonging to the majority class (N instances) is higher than 2/3, we first perform a Random Undersample (RU) until obtaining a representation of 2/3 in the dataset and then used SMOTE^36^ to oversample the minority class examples achieving an equal number of both classes.

### Baseline results: prognostic models based on patient snapshots

Reproducing the methodology based only on patient snapshots and time windows presented by Carreiro et al.^12^, we performed experiments to predict the evolution of a given patient to a critical status for each of the critical endpoints of interest. Predicting the progression to assisted ventilation (need for NIV) is further included. The experiments were conducted with the datasets preprocessed, as explained in previous sections. Resulted from the creation of snapshots, missing values are observed (ranging between 8 and 15% prevalence). To surpass this problem, and since we are dealing with temporal data, we imputed missing values using the values in the previous snapshot (Last Observation Carried Forward). After this, for the snapshots that had not an earlier snapshot (which were residual in number), we imputed missings with the mean/mode for the specific feature.

We evaluated four classifiers: Naive Bayes (NB), SVM with Gaussian kernel, XGBoost (XGB), and Random Forests (RF) due to their state-of-the-art performance in this kind of predictive task^23,25^.

The evaluation was made using a 5 $\times$ 10-fold stratified cross-validation scheme where we ensured that all the assessments from a given patient were all in the train/test fold. Moreover, to improve the model performance, we tackled the class imbalance within cross-validation, applying the same steps explained in the previous section only in the training folds.

Tables 6 and 7 show the benchmark results. Superior results are observed against the reference state-of-the-art results gathered in a previous study (need for NIV)^12^. As observed in the original study^12^, the results for Sensitivity are lower than for Specificity, understandable as positive cases (Evolution = Y) are the minority class.

**Table 6:** Baseline results using data preprocessed following the approach proposed by Carreiro et al.^12^ learned with 4 classifiers: Naive Bayes (NB), Support Vector Machine (SVM), Random Forests (RF) and XGB (eXtreme Gradient Boosting) to predict the Evolution for each of the target endpoints, C1, C2, and C3, within the considered time windows (90, 180 and 365 days), respectively.

	AUC	Sensitivity	Specificity
C1—Need for NIV			
90 days			
NB	77.23 ± 3.55	62.84 ± 7.56	74.30 ± 1.53
SVM	74.06 ± 4.34	71.41 ± 9.55	66.00 ± 3.43
RF	74.97 ± 3.20	57.52 ± 7.85	76.34 ± 1.96
XGB	77.59 ± 2.78	61.70 ± 7.57	77.17 ± 1.77
180 days			
NB	76.23 ± 1.86	63.70 ± 4.41	74.87 ± 1.63
SVM	75.35 ± 1.66	71.32 ± 3.65	66.99 ± 2.33
RF	76.11 ± 1.69	61.08 ± 4.53	76.72 ± 1.88
XGB	76.75 ± 1.70	61.83 ± 4.16	76.16 ± 1.79
365 days			
NB	72.23 ± 1.43	55.96 ± 2.71	74.49 ± 2.34
SVM	71.66 ± 1.49	67.50 ± 2.36	65.33 ± 2.52
RF	78.34 ± 2.03	63.50 ± 3.57	78.11 ± 1.84
XGB	75.60 ± 1.77	61.20 ± 3.00	75.68 ± 1.78
C2—need for an auxiliary communication device			
90 days			
NB	87.69 ± 2.82	81.93 ± 6.24	78.19 ± 1.90
SVM	83.31 ± 4.46	75.28 ± 10.58	73.85 ± 3.06
RF	85.71 ± 3.20	70.14 ± 9.34	81.80 ± 2.19
XGB	86.44 ± 2.93	73.86 ± 7.64	80.87 ± 1.94
180 days			
NB	88.68 ± 1.36	82.94 ± 4.64	78.74 ± 1.15
SVM	89.81 ± 1.45	84.43 ± 4.25	79.76 ± 1.29
RF	89.31 ± 1.03	78.56 ± 4.20	82.94 ± 1.40
XGB	89.53 ± 1.03	81.34 ± 3.53	82.33 ± 1.38
365 days			
NB	86.66 ± 1.83	78.93 ± 3.46	80.22 ± 1.21
SVM	88.13 ± 1.67	81.82 ± 2.78	80.85 ± 1.26
RF	88.28 ± 1.27	75.87 ± 3.94	83.47 ± 1.26
XGB	88.18 ± 1.28	76.59 ± 3.56	82.88 ± 1.33
C3—need for PEG			
90 days			
NB	87.79 ± 2.64	82.33 ± 8.27	80.03 ± 1.77
SVM	84.39 ± 4.70	83.00 ± 13.01	70.19 ± 4.14
RF	86.28 ± 2.92	71.00 ± 12.05	83.91 ± 2.03
XGB	88.32 ± 1.92	77.67 ± 8.41	82.98 ± 2.14
180 days			
NB	88.24 ± 1.71	81.76 ± 5.44	79.09 ± 1.46
SVM	90.30 ± 1.59	85.84 ± 4.83	79.80 ± 1.32
RF	88.59 ± 1.55	75.92 ± 5.95	83.84 ± 1.39
XGB	89.38 ± 1.56	81.04 ± 5.52	83.75 ± 1.27
365 days			
NB	84.82 ± 1.62	76.08 ± 3.90	76.55 ± 1.45
SVM	87.16 ± 1.67	80.21 ± 3.70	78.98 ± 1.48
RF	86.76 ± 1.29	74.28 ± 3.06	82.62 ± 1.34
XGB	86.74 ± 1.47	75.09 ± 3.92	81.76 ± 1.38

**Table 7:** Baseline results using data preprocessed following the approach proposed by Carreiro et al.^12^ learned with 4 classifiers: Naive Bayes (NB), Support Vector Machine (SVM), Random Forests (RF) and XGB (eXtreme Gradient Boosting) to predict the Evolution for each of the target endpoints, C4 and C5, within the considered time windows (90, 180 and 365 days), respectively.

	AUC	Sensitivity	Specificity
C4—need for a caregiver			
90 days			
NB	76.85 ± 3.44	64.00 ± 9.05	72.77 ± 2.31
SVM	72.58 ± 3.71	63.89 ± 6.38	68.66 ± 3.29
RF	75.35 ± 4.08	57.16 ± 8.70	77.13 ± 1.78
XGB	76.10 ± 3.34	57.37 ± 8.36	76.89 ± 2.06
180 days			
NB	79.45 ± 2.06	64.21 ± 3.74	75.93 ± 1.99
SVM	78.63 ± 2.56	72.05 ± 4.84	70.42 ± 2.23
RF	78.89 ± 1.63	64.46 ± 3.70	76.51 ± 2.32
XGB	78.61 ± 1.75	64.81 ± 3.50	76.28 ± 2.25
365 days			
NB	77.61 ± 2.05	58.76 ± 4.18	77.33 ± 2.64
SVM	77.58 ± 2.10	65.22 ± 2.72	74.74 ± 2.81
RF	83.33 ± 1.57	75.05 ± 3.20	76.55 ± 2.41
XGB	80.83 ± 1.43	73.30 ± 2.85	74.07 ± 2.46
C5—need for a wheelchair			
90 days			
NB	80.83 ± 2.92	77.44 ± 8.53	72.16 ± 1.75
SVM	79.32 ± 2.58	73.60 ± 6.60	68.66 ± 2.16
RF	79.65 ± 3.23	64.16 ± 8.04	78.78 ± 2.02
XGB	81.85 ± 2.75	68.48 ± 6.72	77.95 ± 1.96
180 days			
NB	82.19 ± 1.79	73.14 ± 4.57	74.48 ± 1.60
SVM	83.90 ± 1.87	81.80 ± 3.91	71.51 ± 1.76
RF	81.31 ± 1.79	66.55 ± 4.68	79.53 ± 1.53
XGB	82.13 ± 1.75	68.39 ± 4.73	79.56 ± 1.62
365 days			
NB	78.53 ± 1.71	66.26 ± 2.98	74.47 ± 1.45
SVM	81.13 ± 1.97	78.13 ± 3.93	69.66 ± 1.81
RF	82.54 ± 1.64	68.46 ± 3.18	80.41 ± 1.63
XGB	80.87 ± 1.30	66.43 ± 3.53	80.06 ± 1.79

### Triclustering-based classification results

To prove that historical clinical evaluations improve the model predictions, using triclusters as features, we applied our triclustering-based classification approach in accordance with the principles introduced in section “Methods”. For this case study, we opted to detach the triclusters into biclusters and then use them as features. Note that these biclusters are slices of the mined triclusters representing the temporal disease progression. As introduced, each slice is used individually to better represent the state of patients at a specific time point, given the expected differences across the temporal dimension.

As for the baseline, we performed experiments using four classifiers: Naive Bayes, SVM with Gaussian kernel, XGBoost, and Random Forests. The full results are documented in Supplementary Information File SI1 corresponding to the prognostic models for predicting the progression to the critical status *C1, need for NIV*; *C2, need for an auxiliary communication device*; *C3, need for PEG*; *C4, need for a caregiver* and *C5, need for a wheelchair*, respectively. We present the results for AUC, Sensitivity, and Specificity obtained with the models for time windows of 90, 180, and 365 days, identified by the clinicians as clinically relevant. We considered different numbers of historical assessments, creating datasets with 3, 4, and 5 consecutive snapshots (CS). Note that for each dataset (each one with examples with different history sizes) we applied the proposed approach using distances (D) and correlations (C) as the similarity criteria between the patients and the detached biclusters (from triclusters). Table 8 presents a summary of the best-obtained results for each target endpoint according to the three different considered time windows.

**Figure 9:**
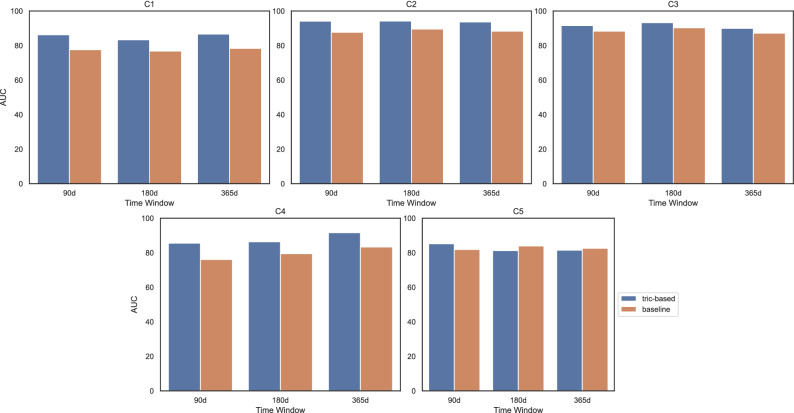
Comparative plot of AUC results obtained by the baseline vs the triclustering-based classifier. Blue bars are referred to triclustering-based classifier results while orange bars are referred to the baseline.

Comparing the gathered results with the baseline obtained by the state-of-the-art approach proposed by Carreiro et al.^12^ (see Fig. 9), we highlight the following:

- triclustering-based classification obtained promising results, predicting all the target endpoints with solid accuracy. The best models achieved AUC results up to 90% predicting the progression for the target endpoints;
- overall, triclustering-based predictors using current-and-past patient’s assessments are better than baseline models using only one evaluation (each snapshot individually) in predicting the progression to a critical status in ALS;
- prognostic models of progression to C5 (wheelchair need) were those with minor differences in results against the baseline;
- predicting progression to C1 – C4 states yield distinctively higher predictive accuracy using the proposed triclustering-based approach against baselines. Mid- and long-term predictions yield differences up to 10pp;
- prognostic models achieved AUC above 90% when predicting the need for an auxiliary communication device (C2), PEG (C3) and caregiver (C4). Most of the best predictions needed 5 appointments, but mid-term prediction for the need for PEG (C3) and short-term prediction for the need for a caregiver (C4) only required 3;
- overall, the distance criteria between patients and triclusters, when compared against peer correlation criteria, yield the best predictive results. The models with the best results were typically learned from a patient history with 5 follow-ups. However, for C2 and C4 needs, short-term prognostics (90 days) yielded better results using only the 3 latest snapshots from patient follow-up;
- the high standard deviation of sensitivity estimates shows the inherent difficulty of predicting the positive class (Evolution=Y);
- the triclustering-based approach allows to collect discriminative patterns of disease progression, promoting better model interpretability in clinical domains.

**Table 8:** Summary of the best AUC results obtained with the triclustering-based classification approach for each of the target endpoints according to each of the considered time windows.

	90 days	180 days	365 days
**C1**	86.24 ± 4.03	83.33 ± 2.71	86.63 ± 3.39
	(XGB; D; 5 CS)	(RF; D; 5 CS)	(RF; C; 5 CS)
**C2**	94.12 ± 3.14	94.14 ± 1.84	93.63 ± 3.23
	(RF; D; 5 CS)	(RF; D; 4 CS)	(RF; D; 5 CS)
**C3**	91.53 ± 5.28	93.23 ± 2.87	89.92 ± 5.38
	(XGB; D; 4 CS)	(XGB; D; 3 CS)	(XGB; D; 5 CS)
**C4**	85.52 ± 4.10	86.35 ± 2.43	91.58 ± 2.36
	(RF; D; 3 CS)	(RF; D; 5 CS)	(RF; D; 5 CS)
**C5**	85.18 ± 5.60	81.23 ± 3.34	81.45 ± 4.92
	(SVM; C; 4 CS)	(RF; D; 5 CS)	(RF; D; 5 CS)

D stands for distance matrices as learning examples, while C stands for correlation matrices. C1, need for NIV; C2, need for an auxiliary communication device; C3, need for PEG; C4, need for a caregiver, and C5, need for a wheelchair.

Some limitations should be noted. First, the approach is focused on dynamic features. Note, nevertheless, that static features can be straightforwardly combined along triclustering-based features for the classification training step. Appendix 1 shows the results of using the static features described in Table 10 together with the triclustering features using the best model parameters and classifiers as shown in Table 8. Second, the triclustering algorithm’s ability to deal with the heterogeneity inherent to this type of data is limited since categorical variables need to entail a denormalization step (nominal variables) or numeric encoding (ordinal variables). Finally, despite the considerably large size of the conducted cohort in light of ALS prevalence, the validation of predictors in international populations is highlighted as a subsequent relevant step.

## Model interpretability

The relevance of a prognostic methodology should be evaluated not only by its predictive performance but also by its guarantees of interpretability. The proposed triclustering-based approach allows us to collect essential patterns of disease progression (used as features of the new space), promoting better model interpretability in clinical domains. In addition, the importance of the input patterns/features for the predictive model can be further recovered to rank the discriminative relevance of the underlying patterns.

To perform the model explainability and identify the more relevant patterns used by the models, the unified SHAP approach^37^ was applied. In particular, we select the KernelExplainer, and TreeExplainer methods, which introduce the possibility of directly measuring local feature interaction effects^38^. The goal is to understand what are the most relevant features, what features appear together, and whether the patterns found are clinically relevant to understand the patient’s progression to the critical endpoints: C1, need for NIV; C2, need for an auxiliary communication device; C3, need for PEG; C4, need for a caregiver and C5, need for a wheelchair.

**Figure 10:**
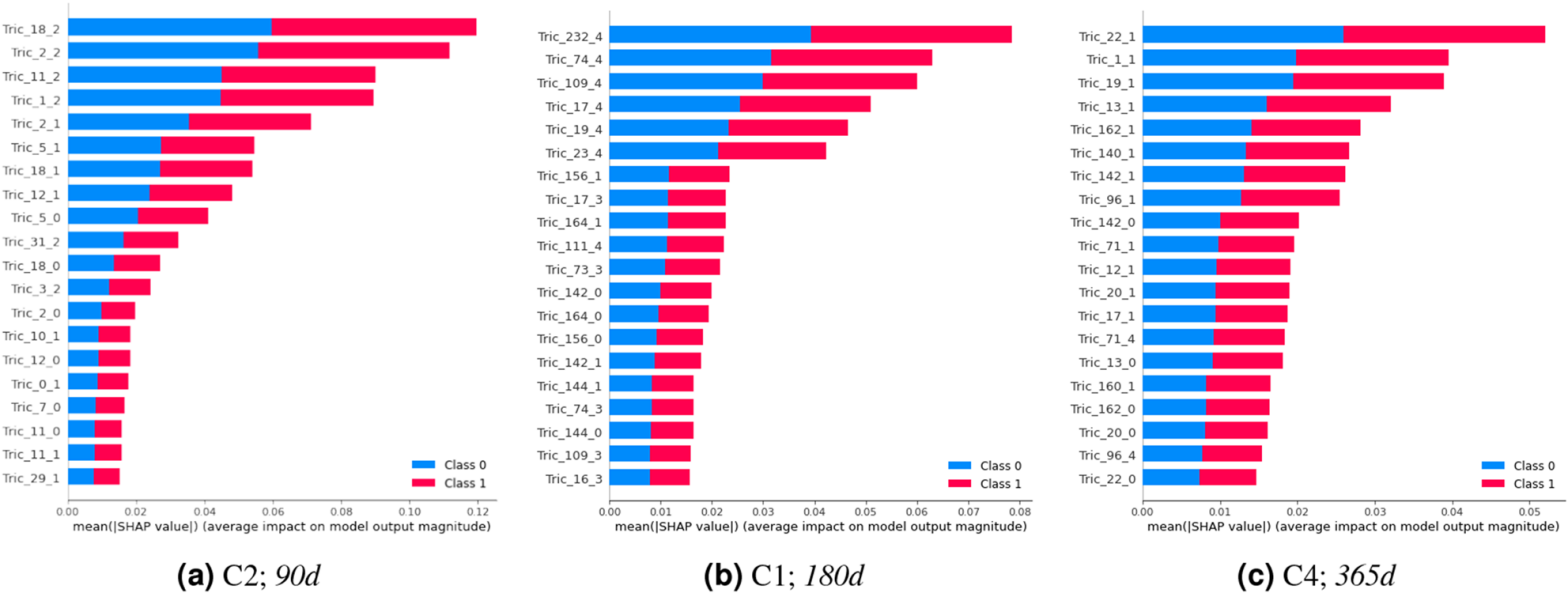
Top 20 patterns (triclusters) used by the triclustering-based classifiers. The terminology used is the following: patterns name starts with ‘Tric’ followed by an identifier, and the snapshot (bicluster) position in the set of snapshots, in which 0 is the first position. Class 0 represents ‘non-evolutions’, and Class 1 represents ‘evolutions’.

**Table 9:** Most relevant patterns used by the best three models.

C2—90 days	
Tric_18_2	[ALSFRSb=12, MITOS-stage=1]
Tric_2_2	[ALSFRSb=12]
Tric_11_2	[ALSFRSb=12, R=12, MITOS-stage=1]
Tric_1_2	[ALSFRSb=12, R=12]
Tric_2_1	[ALSFRSb=12]
C1—180 days	
Tric_232_4	[R=12, MITOS-stage=1]
Tric_74_4	[ALSFRSb=12, R=12, MITOS-stage=1]
Tric_109_4	[ALSFRSb=12, MITOS-stage=1]
Tric_17_4	[ALSFRSb=12, R=12]
Tric_19_4	[R=12]
C4—365 days	
Tric_22_1	[ALSFRSsUL=12, MITOS-stage=1]
Tric_1_1	[ALSFRSb=12, ALSFRSsUL=12]
Tric_19_1	[ALSFRSb=12, ALSFRSsUL=12, MITOS-stage=1]
Tric_13_1	[ALSFRSsUL=12]
Tric_162_1	[ALSFRSsUL=12, MITOS-stage=1]

For simplicity’s sake, we reproduce here only the top 5 patterns. The terminology used is the following: each pattern’s name starts with ‘Tric’ followed by an identifier, and finally, the snapshot (bicluster) position in the set of snapshots, in which 0 is the first position.

We chose to analyze three target endpoints for three different time windows. All the outputs of the remaining endpoints and time windows are made available in a repository (see section “Data availability”). Figure 10 and Table 9 illustrate the top patterns found by TCtriCluster and selected by the classifiers to make the predictions. For the sake of simplicity, we reproduce only the outputs for Random Forest models.

An overall analysis reveals that the majority of the selected patterns refer to the last snapshot/time-point of the triclusters. This makes sense since this is the snapshot closer to the target. However, patterns corresponding to previous snapshots remain relevant since they can reveal other meaningful properties, including the underlying disease progression rate.

## Conclusions

A new methodology was proposed to learn predictive models from longitudinal data using a novel triclustering-based classifier. To this end, TCtriCluster, an extension of triCluster, is proposed to handle heterogeneous clinical data with a temporal contiguity constraint. This restriction was shown to be effective in improving the efficacy of the target predictive models, highlighting its relevance for triclustering three-way time series data. We further show that triclustering-based classification enhances prognostic tasks with the potentialities of model interpretability, enabling the discovery of domain-relevant temporal patterns, then used as features in the predictive models.

As the central case study, we targeted the problem of predicting the clinical progression of ALS patients towards disease endpoints within clinically relevant time windows (90, 180 and 365 days). In particular, we focus on the prognostic of five relevant endpoints (need for non-invasive ventilation, auxiliary communication device, PEG, caregiver, and wheelchair) and assess predictability limits using different lengths of patient historical assessments.

The triclustering-based models achieved good results in short-term predictions (AUC higher than 90%) for the need for an auxiliary communication device and the need for PEG. Short-term prognostics of the need for NIV, caregiver, and wheelchair also yield good predictive performance (AUC around 85%). Some of these models improved their performance while predicting in the mid and long-term. The proposed methodology shows general improvements against state-of-the-art in the capacity to predict the target endpoints, confirming the relevance of using triclusters to perform data transformations sensitive to local patterns of disease progression. The possibility of extracting group-specific patterns along time frames of arbitrary length offers a higher degree of feature expressiveness, which is generally lacking in peer approaches. Another relevant property of the proposed transformation is the preserved interpretability of the produced features as they reveal informative progression patterns that discriminate a given outcome of interest. The inspection of those patterns unravels groups of individuals with coherent temporal variations on a subset of the clinical assessments throughout the follow-up.

This study represents a significant advance in prognostic prediction in ALS, showing generalized improvements in the predictability of degenerative progression towards critical states, meaning clinical interventions. This offers the unique opportunity to better-preparing families for the next illness stages and further entails individualized management with the purpose of optimizing independence, function, and safety, therefore reducing symptom burden and improving the quality of life of the patients.

The proposed triclustering-based methodology can further be used to learn predictive models with different types of three-way data, encompassing prognostic problems in other diseases with available longitudinal cohort studies.

### Supplementary Information

Supplementary Tables.

## Data Availability

The data acquired from the undertaken cohort study are not publicly available to ensure the patients’ rights to privacy and anonymity. Contact the corresponding author for further data access queries. The proposed triclustering-based classifier was coded in Python and is available in https://github.com/dfmsoares/triclustering-based-classifier together with a demo example. The notebooks with model interpretability for all the target endpoints are available in the same repository.

## Appendix 1

### Adding static features

Although the proposed triclustering-based classifier itself does not consider using static features, we decided to add them to the learning matrices to understand if they improve the triclustering-based classifier performance. Table 10 depicts the obtained results with the same parameters that proved to be the best depicted in Table 8. In fact, static features improved the prognostic prediction of some critical points while others remained similar. Table 1 shows the static features used.

**Table 10:** Summary of the best AUC results obtained with the triclustering-based classification approach with static features added for each of the target endpoints according to each of the considered time windows. These results considered the best parameters and classifiers as depicted in Table 8. C1, need for NIV; C2, need for an auxiliary communication device; C3, need for PEG; C4, need for a caregiver, and C5, need for a wheelchair.

	90 days	180 days	365 days
**C1**	87.40 ± 3.83	84.95 ± 2.40	90.07 ± 2.69
**C2**	92.95 ± 3.33	94.59 ± 1.75	93.97 ± 3.09
**C3**	91.41 ± 5.92	93.36 ± 2.76	89.58 ± 5.27
**C4**	87.85 ± 3.76	87.58 ± 2.08	92.81 ± 2.07
**C5**	74.86 ± 6.52	83.24 ± 3.03	82.95 ± 4.91
