# Supplementary material for: Triclustering-based classification of longitudinal data for prognostic prediction: targeting relevant clinical endpoints in amyotrophic lateral sclerosis
Source: Sci Rep. 2023 Apr 15;13:6182. doi: 10.1038/s41598-023-33223-x (PMC10105751; doi:10.1038/s41598-023-33223-x)
Supplement: Supplementary file 1 — Supplementary Tables. [file 41598_2023_33223_MOESM1_ESM.pdf]

Supplementary Information SI1

A Triclustering-based classification in ALS: Full Results

Tables S1, S2, S3, S4, and S5 show the obtained evaluation results corresponding to the prognostic models for predicting the progression to the target endpoints *C1 - Need for NIV*, *C2 - Need for an auxiliary communication device*, *C3 - Need for PEG*, *C4 - Need for a caregiver* and *C5 - Need for a wheelchair*, respectively. We present the results for AUC, Sensitivity and Specificity obtained with these models for time windows of 90, 180 and 365 days. We tried to use different numbers of historical assessments length for each time window: using examples with 3, 4 and 5 consecutive snapshots (CS). Note that for each dataset (each one with examples with different history size) we tried our approach using distances (D) and correlations (C) as similarity between the patients and the detached biclusters (from triclusters).

Table S1. Prognostic prediction of Need for NIV (C1): Results of stratified 5×10-fold cross-validation for time windows of 90, 180 and 365 days (mean value ± standard deviation) using the triclustering-based approach. AUC is the area under the receiver operating characteristics curve. Classifiers are Naive Bayes (NB), Support Vector Machine with Gaussian kernel (SVM), XGBoost (XBG) and Random Forests (RF). Bold-face entries represent the best AUC result for each time window.

Critical 1 - Need for NIV										
		3 CS			4 CS			5 CS		
		AUC	Sensitivity	Specificity	AUC	Sensitivity	Specificity	AUC	Sensitivity	Specificity
90 days										
NB	C	75.75 ± 6.48	67.15 ± 9.37	74.02 ± 8.13	74.16 ± 6.31	56.82 ± 8.79	73.98 ± 7.45	74.47 ± 5.84	59.89 ± 8.78	69.54 ± 8.03
	D	76.30 ± 6.42	63.63 ± 8.58	74.92 ± 7.62	73.64 ± 6.23	64.73 ± 9.21	71.88 ± 7.70	74.75 ± 5.84	65.71 ± 9.42	68.29 ± 7.82
SVM	C	81.10 ± 5.62	68.23 ± 8.18	78.57 ± 8.04	79.41 ± 5.43	79.37 ± 7.13	69.60 ± 7.39	81.85 ± 4.96	88.74 ± 5.40	64.51 ± 6.81
	D	81.91 ± 5.80	71.30 ± 8.28	77.14 ± 7.88	80.95 ± 5.34	79.94 ± 7.25	69.03 ± 7.49	81.88 ± 4.93	90.17 ± 5.30	64.06 ± 6.64
XGB	C	83.38 ± 4.62	80.45 ± 7.08	75.68 ± 8.33	83.64 ± 4.94	80.41 ± 7.68	72.96 ± 7.23	84.29 ± 3.95	80.86 ± 6.40	73.60 ± 7.10
	D	83.39 ± 4.63	79.43 ± 7.46	74.20 ± 7.53	83.35 ± 5.31	79.33 ± 8.10	72.33 ± 8.06	**86.24 ± 4.03**	82.86 ± 6.54	72.91 ± 6.00
RF	C	84.29 ± 5.80	78.01 ± 8.28	75.11 ± 7.88	82.45 ± 5.34	80.07 ± 7.25	72.22 ± 7.49	85.42 ± 4.93	81.43 ± 5.30	74.29 ± 6.64
	D	84.10 ± 4.23	80.17 ± 7.25	74.82 ± 8.36	83.23 ± 4.83	80.06 ± 7.01	71.65 ± 8.62	85.66 ± 4.05	82.63 ± 5.42	74.80 ± 7.81
180 days										
NB	C	71.12 ± 3.57	42.92 ± 5.11	81.98 ± 4.28	73.03 ± 3.19	49.26 ± 4.55	77.59 ± 4.73	75.39 ± 3.43	53.85 ± 5.16	77.69 ± 3.90
	D	71.44 ± 3.40	51.62 ± 5.52	76.67 ± 4.89	72.25 ± 3.48	56.24 ± 4.66	73.74 ± 4.71	74.30 ± 3.43	59.46 ± 4.57	75.02 ± 4.00
SVM	C	72.68 ± 3.97	52.76 ± 4.71	81.01 ± 4.91	75.87 ± 3.32	65.36 ± 4.02	78.35 ± 4.45	78.59 ± 3.97	72.77 ± 4.44	74.70 ± 4.12
	D	75.49 ± 3.47	60.92 ± 4.94	75.19 ± 5.70	78.78 ± 3.39	67.23 ± 3.78	78.14 ± 4.16	79.63 ± 3.69	74.24 ± 4.34	74.40 ± 4.16
XGB	C	77.57 ± 2.55	71.44 ± 3.29	70.27 ± 4.71	80.55 ± 2.88	73.92 ± 4.15	73.34 ± 4.13	82.27 ± 2.72	78.07 ± 4.61	73.89 ± 5.17
	D	78.55 ± 3.02	72.27 ± 4.31	70.85 ± 4.80	80.80 ± 2.97	74.35 ± 3.87	73.90 ± 4.31	82.33 ± 2.81	78.60 ± 3.56	74.34 ± 4.74
RF	C	78.80 ± 3.47	72.25 ± 4.94	70.72 ± 5.70	81.22 ± 3.39	72.88 ± 3.78	73.12 ± 4.16	82.85 ± 3.69	77.40 ± 4.34	75.02 ± 4.16
	D	79.88 ± 2.69	73.48 ± 3.47	71.46 ± 4.73	81.90 ± 2.89	73.47 ± 4.11	74.02 ± 4.21	**83.33 ± 2.71**	77.99 ± 3.75	75.17 ± 4.72
365 days										
NB	C	72.33 ± 3.90	56.19 ± 5.27	81.12 ± 4.63	78.23 ± 4.55	70.50 ± 7.02	76.85 ± 5.55	79.06 ± 4.45	72.55 ± 5.87	78.55 ± 5.69
	D	72.29 ± 3.91	56.82 ± 5.34	77.80 ± 5.32	77.47 ± 4.65	68.87 ± 7.10	77.84 ± 5.12	78.17 ± 4.45	71.96 ± 5.87	77.69 ± 5.87
SVM	C	75.83 ± 4.33	52.17 ± 5.94	88.43 ± 4.08	81.22 ± 3.54	66.24 ± 6.75	86.74 ± 4.19	83.84 ± 3.67	69.80 ± 5.79	89.33 ± 4.71
	D	75.85 ± 4.33	53.62 ± 5.70	87.49 ± 4.04	83.50 ± 3.72	66.91 ± 6.92	85.67 ± 4.45	86.16 ± 3.39	69.88 ± 5.83	87.84 ± 4.80
XGB	C	77.03 ± 3.92	68.06 ± 5.55	75.75 ± 5.31	82.99 ± 3.86	73.93 ± 5.95	80.26 ± 4.85	85.68 ± 3.23	77.73 ± 5.57	82.20 ± 5.29
	D	77.96 ± 3.70	69.66 ± 5.23	75.99 ± 5.04	83.22 ± 3.44	73.40 ± 5.31	80.64 ± 4.40	85.69 ± 3.31	78.16 ± 5.57	82.08 ± 5.80
RF	C	78.00 ± 4.33	67.82 ± 5.70	75.60 ± 4.04	83.46 ± 3.72	73.47 ± 6.92	81.57 ± 4.45	**86.63 ± 3.39**	77.37 ± 5.83	83.92 ± 4.80
	D	78.62 ± 3.32	68.21 ± 6.07	75.93 ± 5.86	83.32 ± 3.78	73.33 ± 5.87	80.50 ± 5.33	86.00 ± 3.47	77.25 ± 6.19	83.06 ± 5.91

Table S2. Prognostic prediction of Need for an auxiliary communication device (C2): Results of stratified 5×10-fold cross-validation for time windows of 90, 180 and 365 days (mean value ± standard deviation) using the triclustering-based approach. AUC is the area under the receiver operating characteristics curve. Classifiers are Naive Bayes (NB), Support Vector Machine with Gaussian kernel (SVM), XGBoost (XBG) and Random Forests (RF). Bold-face entries represent the best AUC result for each time window.

Critical 2 - Need for an auxiliary communication device										
		3 CS			4 CS			5 CS		
		AUC	Sensitivity	Specificity	AUC	Sensitivity	Specificity	AUC	Sensitivity	Specificity
90 days										
NB	C	87.49 ± 4.42	69.96 ± 9.50	86.80 ± 5.74	82.98 ± 5.91	57.49 ± 9.34	85.04 ± 6.86	83.36 ± 5.97	67.30 ± 7.73	79.53 ± 7.80
	D	88.89 ± 4.25	75.51 ± 8.33	86.66 ± 6.47	82.19 ± 6.19	65.29 ± 9.83	81.82 ± 6.66	84.61 ± 5.97	70.94 ± 8.41	81.59 ± 8.41
SVM	C	90.12 ± 3.93	74.66 ± 7.73	90.22 ± 5.30	84.96 ± 5.59	82.75 ± 8.06	77.69 ± 7.15	86.16 ± 5.35	80.65 ± 7.75	79.00 ± 7.59
	D	93.00 ± 3.47	86.83 ± 6.49	84.44 ± 7.54	88.37 ± 4.21	85.84 ± 7.89	79.68 ± 7.09	89.97 ± 4.34	85.20 ± 6.97	76.18 ± 8.08
XGB	C	92.18 ± 3.70	85.71 ± 6.39	85.02 ± 7.16	89.55 ± 4.75	86.46 ± 8.19	81.41 ± 8.82	89.45 ± 4.07	81.75 ± 7.93	79.39 ± 8.55
	D	93.40 ± 3.15	88.24 ± 6.03	86.02 ± 6.64	90.00 ± 4.49	86.77 ± 7.73	81.26 ± 8.50	90.59 ± 4.25	85.44 ± 7.36	82.28 ± 7.54
RF	C	93.61 ± 3.47	86.87 ± 6.49	86.43 ± 7.54	90.92 ± 4.21	85.19 ± 7.89	82.45 ± 7.09	90.95 ± 4.34	81.90 ± 6.97	81.11 ± 8.08
	D	**94.12 ± 3.14**	88.83 ± 5.22	86.96 ± 7.12	91.60 ± 3.99	87.18 ± 6.50	81.41 ± 8.27	92.29 ± 3.62	86.85 ± 7.53	82.47 ± 7.26
180 days										
NB	C	80.14 ± 3.98	50.00 ± 8.53	84.56 ± 4.09	79.28 ± 4.14	55.90 ± 7.16	83.79 ± 4.92	81.36 ± 3.95	59.89 ± 7.78	80.30 ± 5.68
	D	84.18 ± 3.74	66.98 ± 6.82	81.54 ± 4.78	81.39 ± 3.98	60.03 ± 6.88	81.57 ± 4.81	82.18 ± 3.95	64.84 ± 7.35	80.97 ± 5.61
SVM	C	84.79 ± 3.75	63.12 ± 6.47	84.95 ± 4.26	86.48 ± 3.41	67.20 ± 6.32	85.23 ± 4.59	88.09 ± 3.15	79.00 ± 5.61	82.65 ± 4.72
	D	92.63 ± 2.70	90.74 ± 4.17	81.61 ± 4.59	91.82 ± 2.47	90.18 ± 4.10	78.85 ± 5.16	92.61 ± 1.77	87.59 ± 4.73	80.44 ± 4.89
XGB	C	93.27 ± 2.20	88.14 ± 4.69	85.86 ± 3.99	92.98 ± 2.09	88.20 ± 4.93	84.30 ± 4.27	92.30 ± 2.57	86.84 ± 4.86	84.87 ± 4.49
	D	93.54 ± 2.39	89.86 ± 4.04	85.26 ± 4.77	93.64 ± 2.00	88.88 ± 4.03	84.73 ± 4.85	93.03 ± 2.30	87.88 ± 4.72	84.61 ± 4.42
RF	C	93.75 ± 2.70	89.40 ± 4.17	85.89 ± 4.59	93.41 ± 2.47	87.99 ± 4.10	84.73 ± 5.16	92.82 ± 1.77	86.92 ± 4.73	83.97 ± 4.89
	D	94.06 ± 2.10	91.23 ± 4.18	85.72 ± 4.40	**94.14 ± 1.84**	89.03 ± 4.49	85.24 ± 5.24	93.68 ± 2.10	88.26 ± 4.45	85.16 ± 4.36
365 days										
NB	C	80.48 ± 4.42	58.55 ± 7.14	85.17 ± 5.72	80.73 ± 5.01	64.22 ± 7.80	83.11 ± 6.39	81.64 ± 5.39	68.93 ± 9.13	82.39 ± 4.62
	D	81.23 ± 4.18	62.17 ± 7.15	85.83 ± 5.38	82.09 ± 4.64	66.21 ± 7.96	84.87 ± 6.00	82.72 ± 5.39	69.69 ± 9.23	82.01 ± 4.88
SVM	C	86.75 ± 3.12	55.79 ± 7.20	95.98 ± 2.93	86.84 ± 3.83	61.94 ± 7.50	92.55 ± 3.56	89.06 ± 3.91	65.73 ± 8.81	92.78 ± 3.56
	D	90.53 ± 2.67	77.35 ± 6.79	83.26 ± 5.93	90.14 ± 3.23	83.42 ± 5.92	80.73 ± 6.02	92.14 ± 3.20	80.57 ± 8.49	86.17 ± 5.02
XGB	C	90.50 ± 2.70	83.43 ± 5.41	83.66 ± 5.91	92.28 ± 2.74	88.41 ± 5.00	85.00 ± 5.52	92.01 ± 2.95	84.95 ± 5.99	85.84 ± 5.89
	D	90.36 ± 3.01	83.57 ± 5.49	83.71 ± 4.91	92.50 ± 2.91	87.83 ± 5.40	84.25 ± 5.02	93.09 ± 3.14	87.85 ± 5.85	87.13 ± 5.68
RF	C	91.50 ± 2.67	81.87 ± 6.79	83.41 ± 5.93	92.74 ± 3.23	86.48 ± 5.92	83.29 ± 6.02	92.64 ± 3.20	83.12 ± 8.49	85.29 ± 5.02
	D	91.90 ± 2.57	82.78 ± 6.25	83.31 ± 5.53	93.35 ± 2.78	87.83 ± 5.35	83.57 ± 5.18	**93.63 ± 3.23**	87.42 ± 5.86	87.37 ± 5.08

Table S3. Prognostic prediction of Need for PEG (C3): Results of stratified 5×10-fold cross-validation for time windows of 90, 180 and 365 days (mean value ± standard deviation) using the triclustering-based approach. AUC is the area under the receiver operating characteristics curve. Classifiers are Naive Bayes (NB), Support Vector Machine with Gaussian kernel (SVM), XGBoost (XBG) and Random Forests (RF). Bold-face entries represent the best AUC result for each time window.

Critical 3 - Need for PEG										
		3 CS			4 CS			5 CS		
		AUC	Sensitivity	Specificity	AUC	Sensitivity	Specificity	AUC	Sensitivity	Specificity
90 days										
NB	C	73.88 ± 11.25	34.55 ± 13.39	85.04 ± 9.31	77.48 ± 7.78	44.58 ± 14.09	82.41 ± 11.78	82.84 ± 8.25	52.59 ± 14.07	83.38 ± 10.50
	D	79.31 ± 10.48	67.27 ± 13.67	73.65 ± 13.28	80.19 ± 7.82	61.04 ± 14.53	75.19 ± 11.09	84.38 ± 8.25	65.50 ± 14.93	82.45 ± 10.34
SVM	C	78.55 ± 10.91	49.83 ± 13.49	81.82 ± 11.23	81.55 ± 7.42	60.83 ± 14.36	81.17 ± 9.08	85.53 ± 7.13	72.17 ± 13.03	81.10 ± 10.67
	D	86.43 ± 8.02	87.19 ± 8.45	70.54 ± 12.88	88.59 ± 7.55	80.21 ± 10.86	78.42 ± 11.55	86.93 ± 7.31	82.14 ± 11.39	74.05 ± 11.53
XGB	C	83.08 ± 9.40	80.54 ± 11.31	74.09 ± 12.12	84.84 ± 7.47	79.27 ± 12.59	72.60 ± 12.96	86.51 ± 6.83	79.62 ± 10.62	78.67 ± 9.89
	D	86.34 ± 8.55	83.78 ± 11.68	76.56 ± 12.78	**91.53 ± 5.28**	84.54 ± 9.85	82.47 ± 11.89	88.54 ± 6.83	82.77 ± 10.90	79.96 ± 11.24
RF	C	85.68 ± 8.02	83.63 ± 8.45	74.67 ± 12.88	85.67 ± 7.55	77.42 ± 10.86	75.38 ± 11.55	88.57 ± 7.31	81.13 ± 11.39	79.72 ± 11.53
	D	86.93 ± 7.60	88.91 ± 10.43	76.38 ± 11.94	89.92 ± 5.28	86.29 ± 8.85	77.90 ± 10.79	89.31 ± 6.55	82.79 ± 10.26	80.06 ± 10.86
180 days										
NB	C	75.79 ± 5.67	38.09 ± 9.12	82.95 ± 5.45	76.48 ± 6.26	36.80 ± 7.90	84.63 ± 6.36	75.95 ± 6.51	40.12 ± 7.94	83.87 ± 6.56
	D	84.74 ± 4.94	77.60 ± 6.87	79.45 ± 7.01	80.97 ± 5.45	69.21 ± 7.97	76.42 ± 6.90	81.02 ± 6.51	70.59 ± 7.36	74.61 ± 8.72
SVM	C	84.80 ± 3.84	64.71 ± 6.01	81.09 ± 5.08	79.91 ± 5.02	65.81 ± 7.17	74.36 ± 8.54	81.93 ± 5.03	70.40 ± 7.52	74.60 ± 8.02
	D	91.58 ± 3.41	89.51 ± 4.26	79.73 ± 7.08	86.95 ± 3.98	86.75 ± 5.61	74.42 ± 6.81	87.31 ± 4.75	85.35 ± 6.66	75.79 ± 7.36
XGB	C	92.89 ± 3.15	88.20 ± 4.48	82.61 ± 7.25	87.86 ± 3.32	81.95 ± 6.98	76.43 ± 6.50	88.36 ± 4.01	82.32 ± 7.61	78.05 ± 6.90
	D	**93.23 ± 2.87**	88.92 ± 4.51	83.22 ± 6.30	89.68 ± 3.55	83.40 ± 7.92	78.95 ± 6.79	87.49 ± 4.28	84.47 ± 6.43	76.09 ± 7.43
RF	C	92.90 ± 3.41	88.53 ± 4.26	81.97 ± 7.08	88.74 ± 3.98	83.40 ± 5.61	77.33 ± 6.81	88.69 ± 4.75	83.93 ± 6.66	78.41 ± 7.36
	D	93.20 ± 3.00	88.64 ± 4.39	82.68 ± 6.58	90.32 ± 3.16	85.30 ± 7.02	78.11 ± 6.97	89.33 ± 3.59	86.29 ± 7.04	77.93 ± 6.89
365 days										
NB	C	69.08 ± 6.18	31.39 ± 7.50	86.03 ± 5.99	66.52 ± 9.55	27.35 ± 9.94	84.48 ± 8.37	66.37 ± 12.30	26.13 ± 11.30	76.80 ± 13.35
	D	75.53 ± 4.93	58.45 ± 9.46	73.50 ± 7.36	74.04 ± 9.43	49.59 ± 12.40	74.83 ± 10.24	73.46 ± 12.30	53.60 ± 13.59	65.87 ± 12.90
SVM	C	76.42 ± 4.80	54.74 ± 10.19	74.69 ± 7.28	78.67 ± 8.88	44.92 ± 11.31	85.77 ± 9.77	80.30 ± 7.97	44.40 ± 12.12	87.33 ± 8.46
	D	85.28 ± 4.70	79.14 ± 8.30	76.13 ± 8.11	88.01 ± 6.23	84.22 ± 8.25	77.61 ± 8.34	86.51 ± 7.02	78.40 ± 10.80	75.47 ± 11.50
XGB	C	87.90 ± 4.49	82.38 ± 7.43	77.07 ± 8.05	88.78 ± 6.56	85.59 ± 8.17	79.01 ± 10.09	89.80 ± 5.20	82.27 ± 10.55	80.93 ± 9.14
	D	88.72 ± 4.25	84.44 ± 7.06	79.14 ± 8.21	89.57 ± 5.26	85.48 ± 6.87	76.77 ± 9.22	**89.92 ± 5.38**	84.40 ± 7.73	82.27 ± 10.21
RF	C	87.26 ± 4.70	83.57 ± 8.30	76.36 ± 8.11	88.47 ± 6.23	86.08 ± 8.25	75.46 ± 8.34	88.86 ± 7.02	83.20 ± 10.80	79.07 ± 11.50
	D	88.16 ± 4.12	83.81 ± 6.91	76.12 ± 8.49	88.60 ± 5.13	86.51 ± 7.63	74.52 ± 9.19	89.59 ± 5.81	83.33 ± 9.91	78.67 ± 10.33

Table S4. Prognostic prediction of need for a caregiver (C4): Results of stratified 5×10-fold cross-validation for time windows of 90, 180 and 365 days (mean value ± standard deviation) using the triclustering-based approach. AUC is the area under the receiver operating characteristics curve. Classifiers are Naive Bayes (NB), Support Vector Machine with Gaussian kernel (SVM), XGBoost (XBG) and Random Forests (RF). Bold-face entries represent the best AUC result for each time window.

Critical 4 - Need for a caregiver		3 CS			4 CS			5 CS		
		AUC	Sensitivity	Specificity	AUC	Sensitivity	Specificity	AUC	Sensitivity	Specificity
90 days										
NB	C	81.76 ± 3.84	61.11 ± 6.19	80.22 ± 4.69	80.68 ± 3.71	63.50 ± 6.00	79.24 ± 5.51	78.83 ± 4.28	64.88 ± 6.47	77.26 ± 6.34
	D	80.79 ± 3.78	64.27 ± 6.19	79.15 ± 5.14	78.75 ± 3.81	67.64 ± 6.38	77.00 ± 6.12	77.80 ± 4.28	69.85 ± 6.16	73.08 ± 6.20
SVM	C	80.73 ± 4.57	81.11 ± 4.64	75.81 ± 5.71	78.86 ± 4.07	86.32 ± 4.15	67.07 ± 6.29	79.32 ± 4.80	83.16 ± 4.93	65.63 ± 6.61
	D	83.18 ± 3.74	80.39 ± 4.35	76.49 ± 5.84	82.00 ± 3.48	86.43 ± 4.11	67.30 ± 6.31	80.74 ± 4.18	84.23 ± 5.24	66.62 ± 6.25
XGB	C	84.02 ± 4.50	81.52 ± 6.30	77.67 ± 6.23	83.39 ± 3.50	79.51 ± 5.05	76.19 ± 5.39	82.49 ± 3.44	80.19 ± 4.81	74.48 ± 5.89
	D	84.45 ± 3.84	81.83 ± 5.19	77.36 ± 6.85	83.48 ± 3.45	80.61 ± 4.94	76.87 ± 5.22	81.99 ± 3.50	80.50 ± 5.13	73.95 ± 5.99
RF	C	85.45 ± 3.74	81.95 ± 4.35	78.32 ± 5.84	85.25 ± 3.48	79.59 ± 4.11	77.79 ± 6.31	84.06 ± 4.18	79.13 ± 5.24	76.04 ± 6.25
	D	**85.52 ± 4.10**	81.72 ± 5.05	79.31 ± 6.28	85.47 ± 3.00	79.90 ± 5.47	78.02 ± 5.42	84.21 ± 3.53	80.27 ± 5.31	75.24 ± 5.53
180 days										
NB	C	78.51 ± 2.62	51.98 ± 4.74	84.70 ± 2.92	78.84 ± 3.00	54.68 ± 4.62	82.74 ± 3.15	79.89 ± 3.33	67.43 ± 5.55	76.79 ± 4.39
	D	78.10 ± 2.64	58.85 ± 4.72	80.97 ± 3.14	78.23 ± 3.08	60.10 ± 4.59	79.37 ± 3.72	79.84 ± 3.33	68.92 ± 5.63	76.14 ± 4.40
SVM	C	80.50 ± 2.56	61.53 ± 4.81	82.35 ± 3.20	79.95 ± 2.45	72.70 ± 4.09	78.71 ± 3.81	80.66 ± 2.75	82.54 ± 3.86	72.50 ± 4.02
	D	82.68 ± 2.38	68.18 ± 4.47	79.67 ± 3.66	83.16 ± 2.43	73.33 ± 3.96	78.61 ± 3.76	84.23 ± 2.66	83.40 ± 3.84	72.50 ± 4.00
XGB	C	83.21 ± 2.58	77.68 ± 3.77	75.88 ± 4.28	84.25 ± 3.03	79.10 ± 3.44	75.92 ± 4.51	84.26 ± 2.71	81.02 ± 4.69	77.25 ± 3.89
	D	83.70 ± 2.83	77.80 ± 4.03	76.69 ± 4.18	85.21 ± 3.04	80.95 ± 3.18	77.86 ± 4.45	85.17 ± 2.44	81.94 ± 4.22	78.13 ± 4.40
RF	C	84.92 ± 2.38	77.51 ± 4.47	77.15 ± 3.66	85.23 ± 2.43	79.62 ± 3.96	76.79 ± 3.76	85.80 ± 2.66	81.78 ± 3.84	77.88 ± 4.00
	D	85.02 ± 2.27	77.93 ± 4.58	76.57 ± 4.06	85.82 ± 2.42	80.97 ± 3.20	77.16 ± 3.85	**86.35 ± 2.43**	82.50 ± 4.20	78.45 ± 4.27
365 days										
NB	C	81.46 ± 2.66	71.24 ± 4.79	85.23 ± 3.56	85.58 ± 3.02	79.64 ± 5.54	85.59 ± 3.99	86.48 ± 2.83	83.43 ± 4.07	84.98 ± 3.80
	D	81.42 ± 2.73	71.49 ± 4.87	83.97 ± 3.85	85.86 ± 3.11	79.78 ± 5.85	84.16 ± 4.74	86.26 ± 2.83	83.49 ± 4.22	84.55 ± 4.04
SVM	C	81.51 ± 3.22	70.15 ± 5.06	90.49 ± 2.67	84.82 ± 3.99	77.95 ± 5.70	90.31 ± 3.33	88.81 ± 3.56	82.82 ± 4.14	89.11 ± 3.68
	D	85.17 ± 2.80	70.64 ± 5.05	90.54 ± 2.80	87.90 ± 2.94	77.98 ± 5.76	90.28 ± 3.33	90.36 ± 3.21	82.82 ± 4.14	89.14 ± 3.65
XGB	C	86.11 ± 2.97	77.78 ± 4.70	82.86 ± 4.68	89.41 ± 3.03	82.81 ± 5.53	86.16 ± 4.06	89.89 ± 2.77	84.89 ± 4.39	85.70 ± 4.21
	D	86.38 ± 2.45	77.83 ± 4.27	82.24 ± 4.18	89.13 ± 2.85	82.39 ± 4.89	85.31 ± 4.05	90.33 ± 2.86	84.83 ± 4.19	86.19 ± 4.46
RF	C	87.33 ± 2.80	77.09 ± 5.05	84.95 ± 2.80	90.12 ± 2.94	81.07 ± 5.76	86.16 ± 3.33	91.05 ± 3.21	84.10 ± 4.14	87.34 ± 3.65
	D	87.63 ± 2.26	77.14 ± 4.32	84.56 ± 4.14	90.33 ± 2.39	80.87 ± 4.61	86.41 ± 4.03	**91.58 ± 2.36**	84.07 ± 4.30	87.74 ± 4.41

Table S5. Prognostic prediction of need for a wheelchair (C5): Results of stratified 5×10-fold cross-validation for time windows of 90, 180 and 365 days (mean value ± standard deviation) using the triclustering-based approach. AUC is the area under the receiver operating characteristics curve. Classifiers are Naive Bayes (NB), Support Vector Machine with Gaussian kernel (SVM), XGBoost (XBG) and Random Forests (RF). Bold-face entries represent the best AUC result for each time window.

Critical 5 - Need for a wheelchair		3 CS			4 CS			5 CS		
		AUC	Sensitivity	Specificity	AUC	Sensitivity	Specificity	AUC	Sensitivity	Specificity
90 days										
NB	C	73.06 ± 6.80	69.93 ± 9.59	74.92 ± 8.81	77.44 ± 6.26	61.98 ± 8.87	76.22 ± 8.44	75.04 ± 6.39	63.97 ± 9.23	77.27 ± 7.75
	D	73.10 ± 6.89	68.84 ± 8.97	74.50 ± 9.41	76.61 ± 6.67	62.93 ± 8.78	76.70 ± 7.96	74.26 ± 6.39	63.77 ± 9.44	76.51 ± 7.67
SVM	C	80.18 ± 4.64	70.21 ± 7.88	84.58 ± 6.89	**85.18 ± 5.60**	82.53 ± 5.08	79.39 ± 7.16	81.42 ± 5.40	78.14 ± 6.84	79.10 ± 7.65
	D	80.75 ± 4.80	70.41 ± 7.68	84.23 ± 6.96	84.59 ± 5.70	84.31 ± 6.02	79.59 ± 7.11	82.17 ± 6.25	78.76 ± 6.75	79.51 ± 7.79
XGB	C	82.32 ± 5.51	78.63 ± 7.67	77.32 ± 8.58	82.88 ± 6.08	80.62 ± 6.63	80.01 ± 7.61	82.29 ± 4.91	79.51 ± 8.34	76.29 ± 8.33
	D	83.25 ± 5.01	78.36 ± 8.05	77.88 ± 7.39	83.42 ± 5.74	81.37 ± 6.79	79.59 ± 7.62	81.93 ± 5.31	79.65 ± 8.13	75.75 ± 7.95
RF	C	83.90 ± 4.80	78.09 ± 7.68	79.77 ± 6.96	84.87 ± 5.70	82.93 ± 6.02	81.59 ± 7.11	84.68 ± 6.25	79.57 ± 6.75	79.24 ± 7.79
	D	85.04 ± 4.30	78.01 ± 7.60	81.57 ± 6.66	85.14 ± 5.22	82.79 ± 6.95	81.72 ± 7.88	84.55 ± 5.47	79.31 ± 7.69	79.12 ± 7.93
180 days										
NB	C	64.50 ± 4.76	39.15 ± 6.32	78.32 ± 4.19	67.21 ± 4.68	40.09 ± 5.76	78.92 ± 5.12	70.13 ± 3.07	46.69 ± 5.38	75.48 ± 4.27
	D	64.06 ± 4.91	47.66 ± 6.01	71.28 ± 3.80	65.91 ± 4.87	45.03 ± 6.00	74.45 ± 5.26	68.71 ± 3.07	51.66 ± 5.66	74.16 ± 4.47
SVM	C	69.48 ± 4.66	43.16 ± 5.95	81.76 ± 3.48	71.18 ± 4.36	49.15 ± 4.81	81.58 ± 4.43	76.73 ± 2.99	64.82 ± 5.01	77.36 ± 4.35
	D	73.44 ± 4.44	61.96 ± 5.87	73.29 ± 5.32	73.72 ± 3.76	59.36 ± 5.17	74.73 ± 4.09	78.89 ± 2.87	65.82 ± 4.90	78.12 ± 4.74
XGB	C	77.16 ± 3.85	69.27 ± 5.38	70.65 ± 4.87	78.92 ± 2.95	74.55 ± 4.94	70.24 ± 4.87	80.99 ± 3.02	74.68 ± 4.88	74.42 ± 4.51
	D	78.10 ± 3.24	70.23 ± 5.04	71.35 ± 5.20	79.48 ± 2.53	74.32 ± 4.39	70.75 ± 4.94	80.74 ± 3.04	75.22 ± 5.05	74.10 ± 4.27
RF	C	77.47 ± 4.44	71.13 ± 5.87	70.33 ± 5.32	79.01 ± 3.76	73.29 ± 5.17	71.37 ± 4.09	80.59 ± 2.87	72.81 ± 4.90	75.56 ± 4.74
	D	77.84 ± 3.38	70.98 ± 4.71	70.78 ± 5.02	79.27 ± 2.91	72.75 ± 5.21	71.70 ± 4.64	**81.23 ± 3.34**	73.31 ± 5.55	75.55 ± 4.45
365 days										
NB	C	60.93 ± 5.69	35.10 ± 5.81	79.94 ± 4.37	63.84 ± 5.84	44.21 ± 7.57	81.73 ± 4.51	67.80 ± 5.31	50.40 ± 7.63	77.38 ± 6.45
	D	60.24 ± 5.66	36.43 ± 5.95	76.35 ± 5.25	64.02 ± 5.71	46.39 ± 7.65	75.79 ± 5.36	67.05 ± 5.31	51.31 ± 7.81	75.04 ± 6.40
SVM	C	65.52 ± 4.12	29.60 ± 5.74	93.08 ± 3.40	67.94 ± 6.13	37.64 ± 7.75	92.97 ± 3.38	69.95 ± 5.37	42.59 ± 7.43	88.93 ± 4.70
	D	70.50 ± 4.11	45.21 ± 7.08	82.08 ± 5.17	74.04 ± 4.99	41.21 ± 8.21	88.76 ± 4.20	74.98 ± 5.90	45.33 ± 8.28	85.65 ± 6.25
XGB	C	77.06 ± 4.45	72.27 ± 5.94	71.43 ± 6.91	77.64 ± 4.66	69.47 ± 6.97	72.40 ± 6.07	79.97 ± 4.32	73.10 ± 6.13	73.75 ± 6.99
	D	78.07 ± 4.46	73.01 ± 5.86	72.27 ± 5.51	77.93 ± 4.24	69.22 ± 6.79	72.87 ± 6.60	80.99 ± 5.21	75.03 ± 6.46	73.73 ± 6.22
RF	C	76.45 ± 4.11	67.96 ± 7.08	72.34 ± 5.17	77.05 ± 4.99	66.95 ± 8.21	73.00 ± 4.20	80.73 ± 5.90	69.91 ± 8.28	76.12 ± 6.25
	D	77.61 ± 4.35	70.52 ± 6.70	72.69 ± 4.96	77.51 ± 4.33	67.97 ± 7.13	73.68 ± 5.94	**81.45 ± 4.92**	72.38 ± 5.87	76.12 ± 7.29
